# The Tandem PH Domain-Containing Protein 2 (TAPP2) Regulates Chemokine-Induced Cytoskeletal Reorganization and Malignant B Cell Migration

**DOI:** 10.1371/journal.pone.0057809

**Published:** 2013-02-27

**Authors:** Hongzhao Li, Sen Hou, Xun Wu, Saravanan Nandagopal, Francis Lin, Sam Kung, Aaron James Marshall

**Affiliations:** 1 Department of Immunology, University of Manitoba, Winnipeg, Manitoba, Canada; 2 Department of Physics & Astronomy, University of Manitoba, Winnipeg, Manitoba, Canada; 3 Department of Biochemistry and Medical Genetics, University of Manitoba, Winnipeg, Manitoba, Canada; University of Illinois at Chicago, United States of America

## Abstract

The intracellular signaling processes controlling malignant B cell migration and tissue localization remain largely undefined. Tandem PH domain-containing proteins TAPP1 and TAPP2 are adaptor proteins that specifically bind to phosphatidylinositol-3,4-bisphosphate, or PI(3,4)P2, a product of phosphoinositide 3-kinases (PI3K). While PI3K enzymes have a number of functions in cell biology, including cell migration, the functions of PI(3,4)P2 and its binding proteins are not well understood. Previously we found that TAPP2 is highly expressed in primary leukemic B cells that have strong migratory capacity. Here we find that SDF-1-dependent migration of human malignant B cells requires both PI3K signaling and TAPP2. Migration in a transwell assay is significantly impaired by pan-PI3K and isoform-selective PI3K inhibitors, or by TAPP2 shRNA knockdown (KD). Strikingly, TAPP2 KD in combination with PI3K inhibitor treatment nearly abolished the migration response, suggesting that TAPP2 may contribute some functions independent of the PI3K pathway. In microfluidic chamber cell tracking assays, TAPP2 KD cells show reduction in percentage of migrating cells, migration velocity and directionality. TAPP2 KD led to alterations in chemokine-induced rearrangement of the actin cytoskeleton and failure to form polarized morphology. TAPP2 co-localized with the stable F-actin-binding protein utrophin, with both molecules reciprocally localizing against F-actin accumulated at the leading edge upon SDF-1 stimulation. In TAPP2 KD cells, Rac was over-activated and localized to multiple membrane protrusions, suggesting that TAPP2 may act in concert with utrophin and stable F-actin to spatially restrict Rac activation and reduce formation of multiple membrane protrusions. TAPP2 function in cell migration is also apparent in the more complex context of B cell migration into stromal cell layers – a process that is only partially dependent on PI3K and SDF-1. In summary, this study identified TAPP2 as a novel regulator of malignant B cell migration and a potential therapeutic intervention target.

## Introduction

Malignant B cells are characterized by their infiltration and retention in bone marrow and other organs, where they disrupt normal physiological functions, such as hematopoiesis. Leukemia and lymphoma B cells express functional chemokine receptors including CXCR4 and are capable of directional migration (chemotaxis) by following gradients of chemokines such as SDF-1 (CXCL12), the ligand of CXCR4 [1], [2]. Strongly expressed by tissues such as bone marrow, lymph nodes, spleen, lung and liver, SDF-1 is widely known to be an important driving force for the dissemination of cancer cells into these potential destinations [1], [3], [4].

Within bone marrow, SDF-1 attracts cancer B cells into stromal niches that provide survival and proliferation signals and confer resistance to cytotoxic drugs [2], [5]. The interaction of cancer B cells with stromal cells is believed to be a key mechanism accounting for minimal residual disease and relapses after traditional chemotherapy [1], [2]. Therefore, blocking cancer B cell access to and interaction with stromal cells may represent a promising strategy for developing improved therapy.

Evidence has accumulated that the phosphoinositide 3-kinase (PI3K) promotes cancer cell migration [6], [7], [8], [9]. Depending on the cellular context, the PI3K pathway has been proposed to impact migration function at multiple levels, including cell “priming” to enhance overall motility, sensing gradients of chemotactic factors and establishing cell polarity [10], [11]. The major known effector mechanisms involve 3-phosphoinositide messengers produced by PI3K which bind and localize PH domain-containing proteins to the plasma membrane, impacting a variety of cellular functions [9], [12], [13]. The roles of specific 3-phosphoinositides and their binding proteins in cell migration are still not fully resolved.

The tandem PH domain-containing protein 2 (TAPP2), along with its homologue TAPP1, is best known for high-specificity binding to PI(3,4)P2, a phosphoinositide product of PI3K [14], [15], [16]. While the biological functions of PI(3,4)P2 remain to be well understood, several findings suggest that as an effector of the PI3K-PI(3,4)P2 signaling branch, TAPP2 may mediate malignant B cell migration. Previously we found that TAPP2 was predominantly expressed in a more clinically aggressive ZAP-70+ subset of chronic lymphocytic leukemia (CLL) B cells [17], [18], known to be highly migratory in nature [19]. Our study also indicated that in lymphoma and leukemia B cells TAPP2 complexes with components of the dystrophin/utrophin glycoprotein complex (DGC) [17]. Whereas little is known about the functions of the DGC in B cells, it was shown to regulate cell migration in other cell types [20]. Here we provide evidence that TAPP2 controls the migration of leukemia and lymphoma B cell lines in several distinct models dependent on SDF-1. TAPP2 knockdown (KD) impacted on the organization and polarity of the actin cytoskeleton and the activation of Rac1, a key regulator of the actin cytoskeleton and cell migration [21], [22]. Interestingly, however, TAPP2 co-localizes with utrophin at the sides and rear of migrating cells and functions in migration even under conditions where PI3K signaling is inhibited. Our results provide the first evidence that TAPP adaptors function in cell migration and provide initial insights regarding the role of TAPP2 in cytoskeletal reorganization.

## Materials and Methods

### Cell Culture and Lentivirus-mediated Protein Knockdown

Human leukemia or lymphoma B cell lines NALM-6 (DSMZ, Germany), RS4;11 (DSMZ) and RAJI (DSMZ) and bone marrow stromal cell line M2-10B4 (ATCC) were cultured in RPMI1640 (Life Technologies) containing 10% FBS. The bone marrow stromal cell line S17 was obtained from Ken Dorshkind (UCLA) [23] and cultured in Opti-MEM I (Life Technologies) supplemented with 10% FBS. TAPP2 knockdown (KD) in these B cell lines was performed, essentially following previously published protocols [17]. Briefly, lentivirus packaged from the pSIH1-H1-copGFP vector (Systems Biosciences) expressing TAPP2-targeting shRNA (GCUGGAAACGUCGCUUCUUUG) was used to transduce B cell lines at MOI of 5-10. TAPP2 protein KD was verified by Western blot, performed as previously described [17]. TAPP1 protein expression was not found to be affected in TAPP2 KD cells in Western blot using a TAPP1 antibody provided by D. Alessi. To determine cell viability, cells were stained with APC-Annexin V antibody (BD Biosciences) and DAPI (Sigma-Aldrich) and analyzed using a BD FACS Canto II flow cytometer, essentially following a previously established protocol [24]. To examine TAPP2 KD effect on CXCR4 expression, cells were stained with APC mouse anti-human CXCR4 (BD Biosciences) and mean fluorescence intensity (MFI) was measured by flow cytometry.

### Transwell Chamber Chemotaxis Assay

Chemotaxis assays were performed largely as described [25], based on 24-well Transwell chambers with polycarbonate inserts with 8 µm pore size (Corning life Sciences). 5×10^5^ cells suspended in 100 µl migration media and 600 µl of 100 ng/ml SDF-1 (Peprotech) diluted in migration media (RPMI1640 with 0.5% BSA) were used in the top and bottom chambers respectively. After 4–5 hour incubation at 37°C and 5% CO_2_, cells that migrated into the bottom chamber as well as input cells were counted by flow cytometry. Cell migration was defined as the percentage of total input cells that migrated. PI3K inhibitors GDC-0941 (pan), CAL-101 (delta-selective), AS-605240 (gamma-selective) or TGX-221 (beta-selective) were all from Selleck Chemicals and used at 2 µM (pre-applied to input cells and added to transwell chambers). Where indicated, 100 µl of fibronectin (Sigma-Aldrich) or laminin (Sigma-Aldrich), diluted in coating buffer at 10 µg/ml, was used to coat the polycarbonate surface of the Transwell insert at 4°C overnight. The insert was washed twice with RPMI1640 prior to performing migration assay as described above.

### Cell Migration in Microfluidic Chambers

A previously reported, a Y shaped microfluidic device design was used to observe cell migration behavior in a stable chemokine gradient [26]. The microfluidic channel was coated with fibronectin and blocked with BSA. The device was maintained at 37°C by attaching a transparent heater to the back of the coverslide (Thermal-Clear Transparent Heater, Model No. H15227, Minco, MN). Cells were loaded into the microfluidic device and allowed to settle. Medium and chemokine solutions were infused into the device at a total flow rate of 0.2 µl/min to produce a stable chemokine gradient inside the microfluidic channel. The chemokine gradient was confirmed by measuring the fluorescence intensity profile of FITC-Dextran inside the microfluidic channel. Cell migration was recorded by time-lapse microscopy for 1–4 hours. Movement of individual cells was tracked in ImageJ software using the “Manual Tracking” plug-in. The cell tracks were plotted using ibidi analysis software (Chemotaxis and Migration Tool, ibidi LLC, WI). Following previously established analysis methods, the movement of cells was quantitatively evaluated by measuring (1) % of migrating cells, which is defined as the percentage of cells that migrated at least one cell length over the time-lapse experiment; (2) the Chemotactic Index (C.I.), which is the ratio of the displacement of the migrating cell toward the chemokine gradient (Δy), to the total migration distance of the cell (d) using the equation C.I. = Δy/d, presented as the average value ± SEM; (3) the average speed (V) calculated as d/Δt, where Δt is the period of the time-lapse experiment, and presented as the average value ± SEM of all migrating cells.

### Quantification of F-actin by Flow Cytometry

10^6^ cells were resuspended in 400 µl pre-warmed (37°C) cell migration media (RPMI 1640+0.5% BSA) and SDF-1 was added to a final concentration of 100 ng/ml. At the indicated time points post-stimulation, cells were fixed by mixing with 800 ul ice-cold PBS containing 4% paraformaldehyde and incubating on ice for 30 min. After two washes with cold PBS, cells were permeablized with cold PBS containing 1% FBS and 0.1% Trition X-100, and kept on ice for 10 min. Following two washes with cold PBS containing 1% BSA, cells were stained at room temperature with 200 µl of 165 µM Alexa Fluor 647 Phalloidin (Life Technologies) diluted in PBS containing 1% BSA. Cells were washed twice with cold PBS. F-actin level was determined as MFI from flow cytometry analysis.

### Intracellular Staining and Confocal Microscopy

Lab-Tek chambered coverglass (Thermo Scientific) were coated with 10 µg/ml fibronectin at 4°C overnight and washed with RPMI-1640. 1×10^5^ NALM-6 cells were added and incubated for 1–1.5 h to allow adhesion. The supernatant was replaced with 100 ng/ml SDF-1 to stimulate cells for desired time. To fix cells, supernatant was immediately replaced with cold PBS containing 2% paraformaldehyde. Cells were fixed on ice for 30 min on ice. After wash twice with cold PBS, cells were permeabilized with cold PBS containing 1% FBS and 0.1% triton X-100 for 10minutes on ice, followed by wash with cold PBS containing 1% BSA. To examine the localization of utrophin or TAPP2 (relative to F-actin), these proteins were stained largely as previously described [17], using primary antibodies (rabbit polyclonal IgG) against utrophin (Santa Cruz, H-300) or TAPP2 [17] and Alexa Fluor 647-labelled goat anti-rabbit IgG secondary antibody (Life Technologies). This was followed by F-actin staining with Alexa Fluor 488 phalloidin. To assess the colocalization of utrophin and TAPP2, permeabilized cells were first stained for utrophin using the antibody H-300 and Alexa Fluor 488-labelled goat anti-rabbit IgG (Life Technologies), and then stained for TAPP2 using TAPP2 antibody [27] directly labeled with Alexa Fluor 647. The direct labeling was performed using Alexa Fluor 647 Protein Labeling Kit (Life Technologies) following the supplier’s protocol. Imaging was performed using an Olympus IX81/Ultraview LCI confocal microscope as previously described [17]. Correlation coefficient of subcellular localization was quantified with Ultraview software.

The localization of myosin II and active Rac was examined in a similar procedure. Myosin II was stained with rabbit anti non-muscle myosin II heavy chain primary antibody (Covance) and Alexa Fluor 488-labelled goat anti-rabbit IgG secondary antibody (Life Technologies). Active Rac was probed with GST-tagged PAK-PBD protein (Cytoskeleton, Inc.) and GST antibody labeled with Alexa Fluor 647 (26H1, Cell signaling Technology) or HiLyte Fluor 647 (AnaSpec). The two GST antibodies showed the same staining patterns, and did not produce nonspecific signal in control staining where GST-tagged PAK-PBD was omitted.

### Rac and Rho Activation Assays

Active GTPases were pulled down using PAK-PBD-agarose beads (Rac) or Rhotekin-RBD-agarose beads (Rho), according to the manufacturers instructions (EMD Millipore). Western blotting analysis of total and GTP-bound fractions used Rac1 and RhoA/B/C-specific antibodies from EMD Millipore and followed our previously established protocols [28].

### Leukemic Cell Migration into Bone Marrow Stromal Cell Layers (Under-stroma Migration)

2 × 10^5^ NALM-6 cells were seeded onto a confluent layer of S17 or M2-10B4 stromal cells cultured in 24-well plates and incubated for 10 hours at 37°C and 5% CO2. The wells were vigorously washed twice with media to remove leukemic cells that had not migrated into the stromal layer. The stromal layer containing migrated B cells was photographed using phase-contrast microscopy. To quantify the migration, the remaining cells after wash were detached with trypsin/EDTA (Life Technologies), immediately washed and resuspended in RPMI1640 with 10% FBS, followed by staining and counting with flow cytometry. NALM-6 cells were distinguished from stromal cells through a combination of size (forward scatter), granularity (side scatter) and CD19 staining with APC mouse anti-human CD19 antibody (BD Biosciences). Under-stroma migration was defined as the percent of total input cells that migrated. To assess the effect of blocking SDF-1/CXCR4 signaling on this process, NALM-6 cells were pre-incubated for 30 minutes with AMD3100 (Sigma-Aldrich, 10 or 100 µM), SDF-1 (0.1 or 1 µg/ml), pertussis toxin (Sigma-Aldrich, 0.1 or 1 µg/ml) or GDC-0941 (2 µM). This NALM-6 culture was then used to replace the culture supernatant of stromal cells and under-stroma migration was assayed as described above. In lentiviral shRNA transduction experiments, partially transduced NALM6 cell cultures were used and cells recovered from stromal cell layers were further gated as GFP+ or negative. Data were then calculated as percent migration of control or TAPP KD shRNA transduced cells (both GFP+) relative to the GFP negative untransduced NALM-6 population, which provides an internal control.

## Results

### TAPP2 Knockdown Impairs SDF-1-dependent Migration in Transwell Chambers

**Figure 1:**
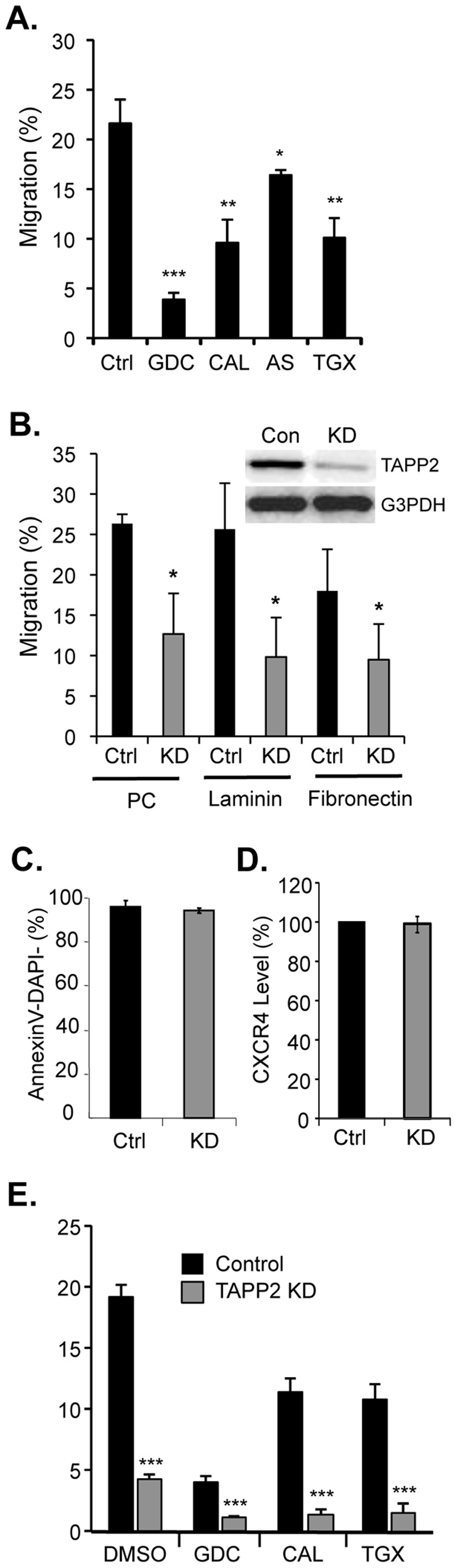
TAPP2 knockdown impairs SDF-1-induced migration. A NALM-6 cells migration towards SDF-1 is impaired by PI3K inhibitors. NALM-6 cells were treated with drug vehicle (DMSO) or PI3K inhibitors GDC-0941, CAL-101, AS-605240 or TGX-221 (all 2 µM), and migration was assayed in transwell chambers containing 100 ng/ml SDF-1. Graphs represent the mean ± SD from three independent experiments. All PI3K inhibitors significantly reduced the migration compared to vehicle control according to Student’s *t* test: *p<0.05, **p<0.01 and ***p<0.001. **B.** TAPP2 knockdown (KD) inhibits NALM-6 migration on a variety of substrata. NALM-6 cells were transduced with control pSIH1-H1-copGFP lentivirus (Con) or virus expressing human TAPP2-targeting shRNA (KD). Western blot represents one of four batches of transduction confirming TAPP2 KD. Transwell assays of migration to 100 ng/ml SDF-1 were performed, where the insert was either uncoated polycarbonate (PC) or coated with 10 µg/ml laminin or fibronectin. Results of three independent experiments are shown as mean ± SD. Significance comparing control versus KD cell migration was calculated for each coating condition using Student’s *t* test: *p<0.05. Note the effect of TAPP2 KD on migration in uncoated transwell chambers has been reproduced in 5 additional experiments. **C.** TAPP2 KD does not significantly affect cell viability. Control or TAPP2 KD NALM-6 cells were stained for Annexin V and with DAPI and analyzed by flow cytometry. Percentages (mean ± SD) of viable cells (Annexin V and DAPI double negative) from four independent experiments are shown. Student *t* test found no significant difference in cell viability. **D.** TAPP2 KD does not significantly affect surface expression of CXCR4. Cells were stained for surface CXCR4 and analyzed by flow cytometry. CXCR4 levels were expressed as percentage of mean fluorescence intensity (MFI) relative to control cells. Bars represent mean ± SD of two independent experiments. **E.** TAPP2 KD inhibits SDF-1-induced migration in combination with PI3K inhibitors. Control or TAPP2 KD NALM-6 cells were assayed for migration to SDF-1 in the presence of vehicle (DMSO) or PI3K inhibitors (2 µM) GDC-0941, CAL-101 or TGX-221. Results are mean ± SD of three independent experiments. Significance of difference in migration was confirmed by Student’s *t* test comparing PI3K inhibitor alone versus the corresponding TAPP2 KD+PI3K inhibitor group: ***p<0.001. Note all TAPP2 KD+PI3K inhibitor groups were also significantly different than TAPP2 KD alone (all p<0.01 by Student’s *t* test).

The potential function of TAPP2 in cell migration was assessed in B cell lines NALM-6 and RS4;11 (derived from human leukemia) and RAJI (human lymphoma), which migrate to SDF-1 in Transwell assay (Fig. 1 and Fig. S1). Considering that TAPP2 is currently defined as a PI3K-regulated adaptor protein with specific binding to PI(3,4)P2, we first tested if the migration of these cell lines depends on PI3K. A variety of PI3K inhibitors were applied to the migration assay, including GDC-0941 (all isoforms), CAL-101 (delta-selective), AS-605240 (gamma-selective) and TGX-221 (beta-selective). These inhibitors all reduced cell migration to SDF-1 (Fig. 1A and Fig. S1A), confirming the PI3K-dependence of cell migration in these cells and suggesting that multiple PI3K isoforms are involved. We then performed TAPP2 gene silencing in these cells. As a result of TAPP2 protein knockdown (KD), cell migration to SDF-1 was significantly inhibited (Fig. 1B and Fig. S1B), providing the first evidence that TAPP2 plays a role in cell migration. TAPP2 KD cells consistently displayed impaired migration on polycarbonate transwell membranes with or without fibronectin or laminin coating (Fig. 1B). TAPP2 KD did not affect cell viability (Fig. 1C) or CXCR4 expression level (Fig. 1D).

Since TAPP2 is considered a PI3K-regulated adaptor protein, we reasoned that the effect of TAPP2 KD would depend on PI3K activity and thus TAPP2 KD would have no further effect when PI3K activity is inhibited. To test this hypothesis, we determined the effect of combined PI3K inhibition and TAPP2 KD on SDF-1-induced migration. Strikingly, while PI3K inhibitors only partially blocked migration, TAPP2 KD in combination with PI3K inhibitor treatments nearly abolished the migration response (Fig. 1E), suggesting that TAPP2 may contribute some functions independent of the PI3K pathway. Basal migration in the transwell assay, seen without chemokine addition, was also sensitive to PI3K inhibitors and was reduced in TAPP KD cells; however in this case no additional reduction was observed with combined treatment (Fig. S1C). Together these results indicate that TAPP2 functions in malignant B cell migration, potentially via both PI3K dependent and independent mechanisms.

### TAPP2 Knockdown Alters Migratory Behavior in a Stable Chemokine Gradient

**Figure 2:**
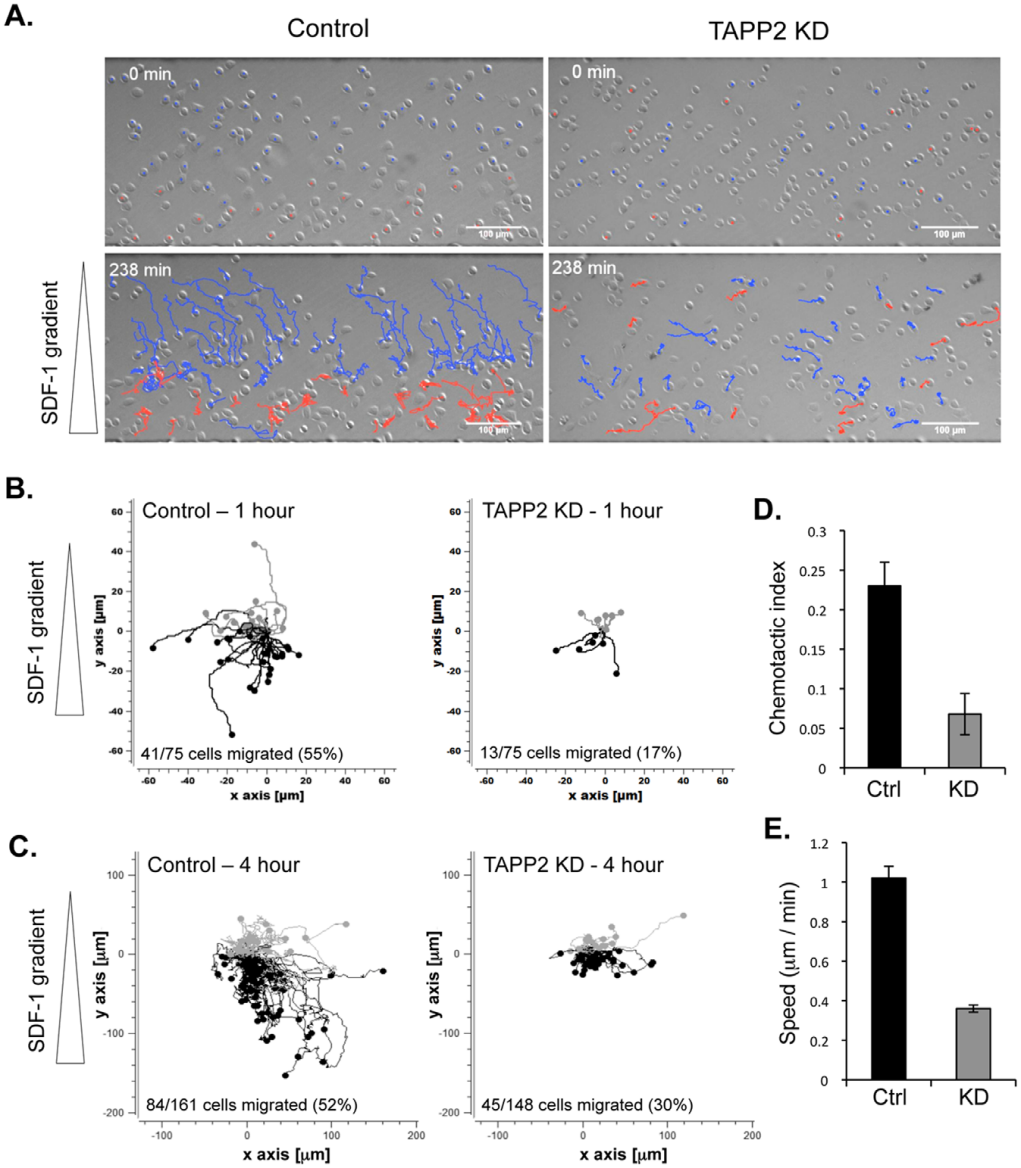
TAPP2 KD leads to decreased migration speed and directionality in a stable SDF-1 gradient. NALM-6 cells transduced with control or TAPP2 KD lentivirus were loaded onto a microfluidic chemotaxis device and exposed to a 100 nM SDF-1 gradient (represented with triangles). Time-lapse images were taken and cell movement was analyzed using cell-tracking software. **A.** Panels show the first and last image from a representative experiment. Migrating cell tracks are marked in blue (migrating toward higher SDF-1 concentration) or red (migrating towards lower SDF-1 concentration). **B.** Migration tracks of control or TAPP2 KD cells in a 1 hour experiment were normalized to a common origin (0, 0) and plotted to visualize patterns of movement in two dimensions (the chemokine gradient is in the y dimension as indicated). Black tracks represent cells migrating toward the gradient and grey tracks are for cells migrating against the gradient. The solid circles indicate the end of the cell tracks. **C.** Migration tracks of control or TAPP2 KD cells in a 4 hour experiment were plotted as in C. **D.** Chemotaxis index of control and TAPP2 KD cells, calculated as described in Methods. **E.** Migration speed of control and TAPP2 KD cells, calculated as described in Methods. Data are representative of four independent experiments.

To further characterize the role of TAPP2 in controlling migratory behavior of malignant B cells, we performed microscopic cell tracking in the presence of a stable chemokine gradient established using microfluidic chambers [26]. TAPP2 KD or control cells were placed in fibronectin-coated chambers and cell movement was recorded for 1–4 hours (Fig. 2A, Video S1 and Video S2). Cell tracks were analyzed to determine the percentage of migrating cells, the migration directionality (chemotactic index) and speed. After 1 hour in the chamber, 55% of control cells migrated at least one cell length, whereas only 17% TAPP2 KD cells migrated (Fig. 2B). By 4 hours, significantly more TAPP KD cells had migrated (30% versus 52% of control cells; Fig. 2C). However TAPP2 KD cells exhibited significantly reduced migration speed and directionality over this time period (Figs. 2D and 2E), indicating that both gradient sensing and motility functions are impaired.

### TAPP2 Regulates Chemokine-Induced Cytoskeletal Rearrangement

**Figure 3:**
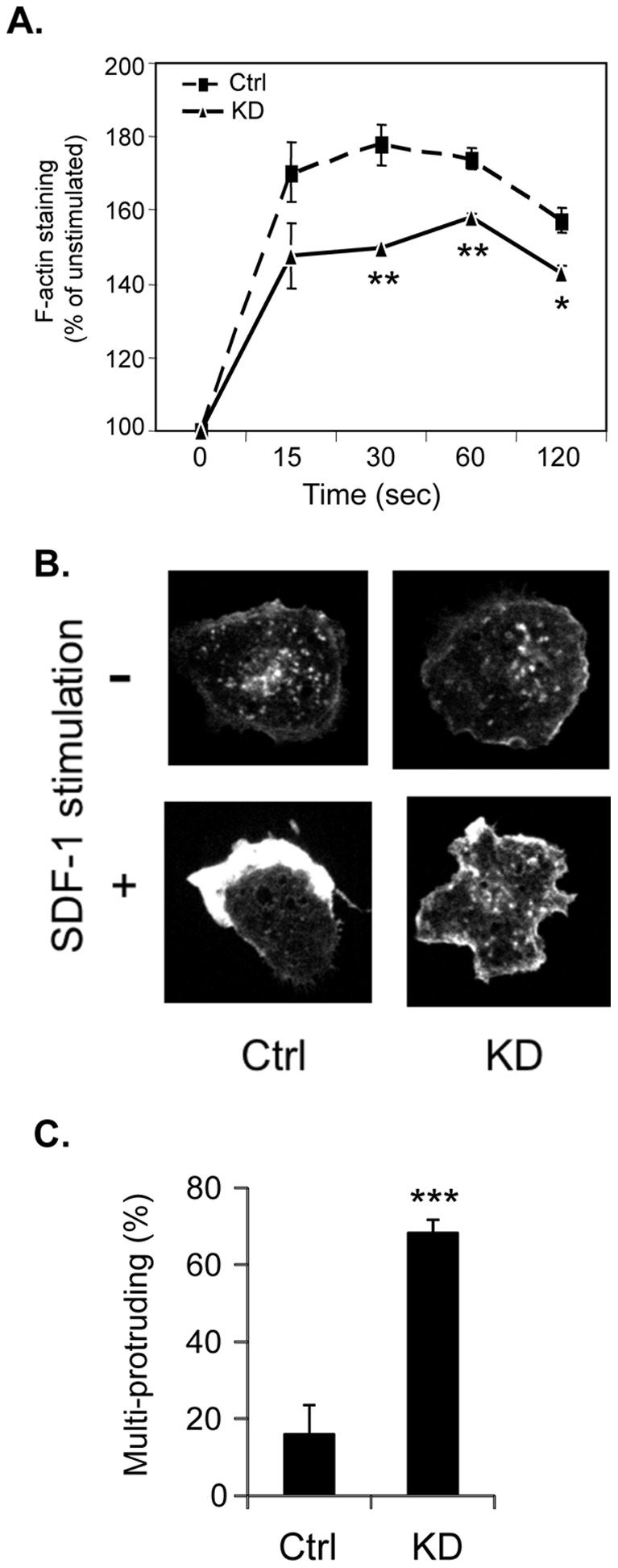
TAPP2 KD impairs chemokine-induced rearrangement of the actin cytoskeleton. **A.** TAPP2 KD cells show impaired accumulation of F-actin in response to SDF-1 stimulation. Control or TAPP2 KD NALM-6 cells were stimulated with 100 ng/ml SDF-1 for a series of time points ranging from 15–120 seconds. Control samples without addition of SDF-1 were used for “time point 0”. Cells were fixed immediately following stimulation, permeabilized, stained with fluorescent phalloidin and mean fluorescence intensity (MFI) was determined by flow cytometry. Results were normalized relative to the level at time point 0. The values were shown as mean ± SEM for three experiments based on 2 batches of transductions. Significance for KD cells versus control cells at each stimulation time point was confirmed by Student’s *t* test: **p < 0.01; *p < 0.05. **B.** TAPP2 KD leads to altered organization of the F-actin cytoskeleton. Cells plated on fibronectin-coated chambered coverglass were stimulated with or without 100 ng/ml SDF-1 for 1 minute, fixed, permeabilized and stained for F-actin using Alexa Fluor 647 phalloidin. Confocal microscopy was performed using a 60× objective. Representative images are shown indicating polarized F-actin distribution in SDF-1-stimulated control cells and multiple cellular protrusions with less intense accumulations of F-actin in TAPP2 KD cells. **C.** Frequency of cells having more than 2 protrusions in SDF-1-stimulated control versus TAPP2 KD cells. Results are presented as mean percentage of cells scored as multi-protrusion ± SD from four independent experiments. For each group more than 400 cells were analyzed. The multi-protruding cell frequency is significantly higher in TAPP2 KD cells than in control cells, as indicated by Student’s *t* test: ***p<0.001.

A prerequisite feature of cell migration is the rearrangement and polarization of the actin cytoskeleton. Given the association of TAPP2 with actin-binding proteins [17], [29], [30] we asked whether TAPP2 KD impacts on migration through disrupting chemoattractant-induced changes in the actin cytoskeleton. SDF-1 stimulation of control cells led to a rapid (within 1 minute) and intense accumulation of filamentous actin (F-actin), as detected by fluorescent phalloidin staining. TAPP2 KD cells also exhibited increase in F-actin after SDF-1 stimulation, however, the intensity of the response was significantly reduced (Fig. 3A). We next visualized the effect on F-actin distribution patterns using confocal microscopy. SDF-1 stimulated control cells showed intense F-actin staining around the circumference of the cell, with a subpopulation exhibiting asymmetric localization within membrane protrusions formed at one end of the cell (Fig. 3B). In contrast, a distinct pattern of actin cytoskeleton organization was seen in the TAPP2 KD cells, which often extended numerous protrusions around the cell periphery staining less intensely for F-actin (Fig. 3B). Scoring of cell morphology indicated a significantly higher frequency of this multi-protrusion morphology in TAPP2 KD cells (Fig. 3C). These results demonstrated that TAPP2 plays a role in cytoskeletal reorganization in response to chemoattractant stimulation.

### Localization of TAPP2 Relative to F-actin and Utrophin

**Figure 4:**
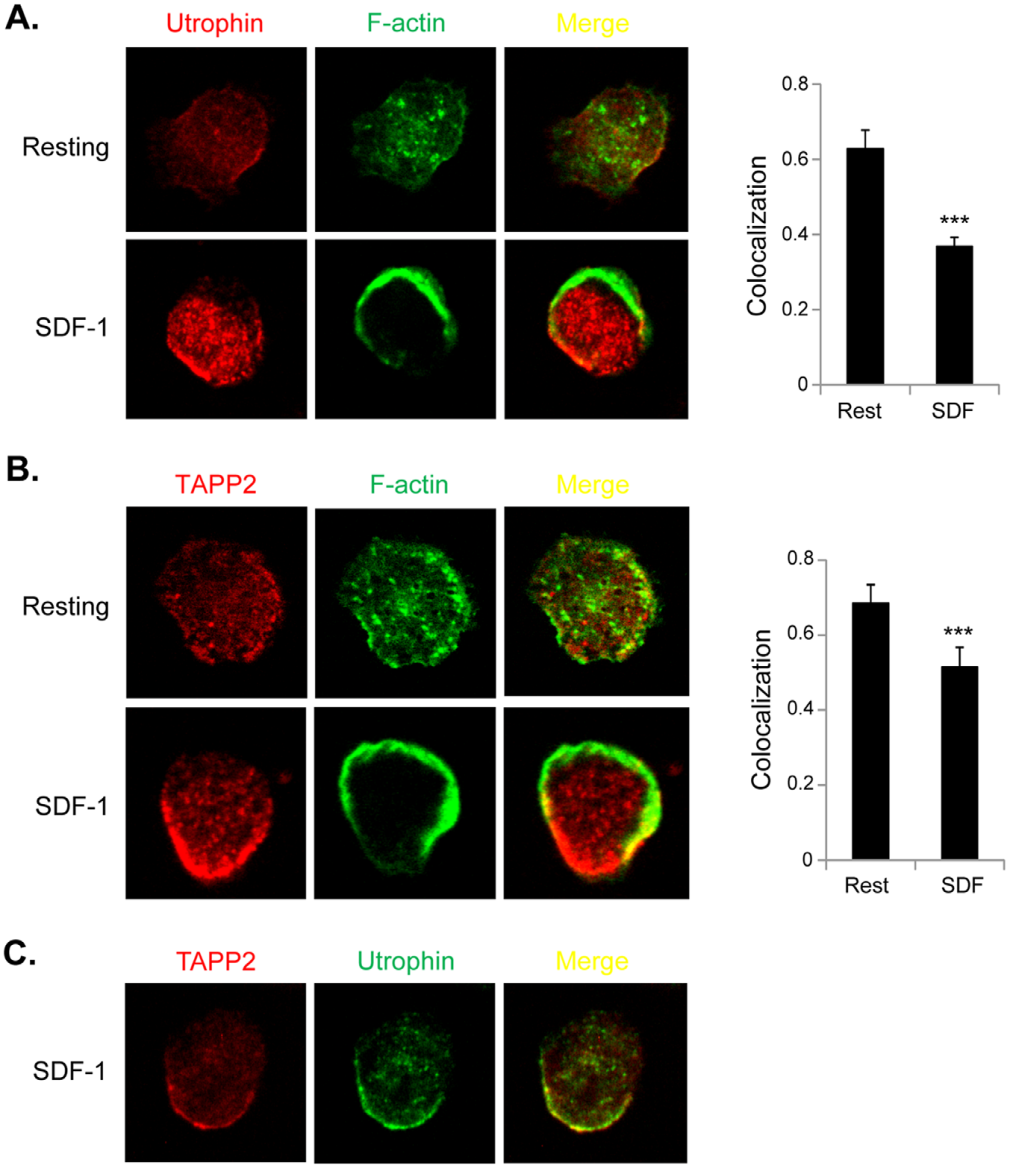
Relative localization of TAPP2, utrophin and F-actin upon chemokine stimulation. A. Relative localization of utrophin versus F-actin. NALM-6 cells without or with 100 ng/ml SDF-1 stimulation for 1 minute were fixed, permeabilized and stained for utrophin and F-actin. Images shown are representative of five experiments. Right panel: Colocalization of utrophin and F-actin was calculated as the average Pearson’s correlation coefficient of more than 100 cells from 5 experiments. With SDF-1 stimulation, the overall colocalization of utrophin and F-actin significantly decreased, as determined by Student *t* test: ***p<0.001. **B.** Relative localization of TAPP2 versus F-actin was visualized and quantified using similar methods as in A (5 experiments). A significant reduction in the overall colocalization of TAPP2 and F-actin was seen in SDF-1 stimulated cells (Student *t* test: ***p<0.001). **C**. TAPP2 colocalizes with utrophin at the sides and rear of migrating cells. NALM-6 cells were stimulated, processed and visualized as in **A**. Images representing two experiments are shown.

TAPP2 was previously found to associate with the actin-binding protein utrophin [17], [29]. We investigated the subcellular localization of TAPP2 relative to F-actin and utrophin. Utrophin is reported to selectively bind a stable population of F-actin via its calponin homology domain [21], [29], [31], whereas the F-actin at the leading edge of migrating cells is rapidly polymerized and depolymerized [32], [33] and does not bind to utrophin. Consistent with the literature, we observed a reciprocal distribution between polarized F-actin and utrophin in SDF-1 stimulated cells (Fig. 4A). We quantified the relative colocalization between F-actin and utrophin by calculating their colocalization coefficient before and after SDF-1 stimulation. The F-actin/utrophin colocalization coefficient was significantly lower in SDF-1 activated cells (Fig. 4A, right panel), consistent with exclusion of utrophin from the dynamic F-actin generated by chemotactic stimulus. We examined the sub-cellular distribution pattern of TAPP2 and found that it also reciprocally localized against the intense dynamic F-actin population and showed a lower coefficient of colocalization with total F-actin in SDF-1-activated cells than in resting cells (Fig. 4B). Consistent with these patterns, TAPP2 and utrophin highly colocalized with each other (Fig. 4C). In summary, the imaging data indicated that TAPP2 localizes together with utrophin and stable F-actin, but away from dynamic F-actin forming at the leading edge.

### TAPP2 KD Led to Dysregulation of Rac

**Figure 5:**
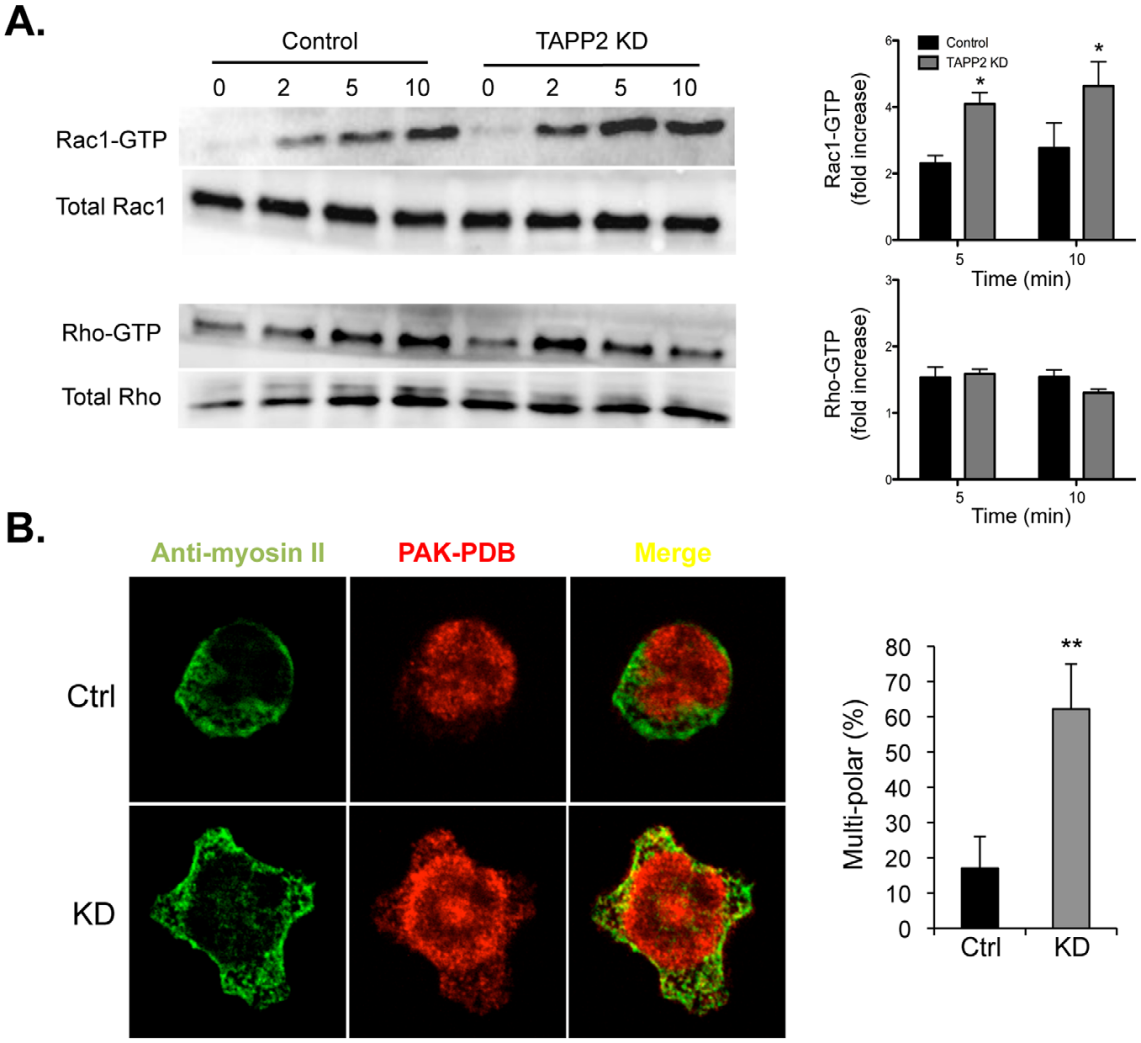
TAPP2 KD leads to dysregulation of Rac. A. TAPP2 KD cells increased activation of Rac, but not Rho. Control or TAPP2 KD NALM-6 cells without stimulation (time point 0) or stimulated with 100 ng/ml SDF-1 for 2, 5 or 10 minutes were lysed. Total protein extracts, Rac-GTP pulldowns or Rho-GTP pulldowns were Western blotted and probed for Rac or Rho. Blots shown represent three independent experiments that all demonstrated a general pattern of Rac overactivation. Activity was quantified as the ratio of active to total Rac or Rho and normalized relative to time point 0. Bar graph on the right indicates the mean and SEM of three independent determinations. *indicates p<0.05 by paired T test. **B.** TAPP2 KD leads to an altered pattern of staining with PAK-PBD and anti-myosin II. Cells were stimulated with SDF-1, fixed and stained with antibody against non-muscle myosin II heavy chain, and with PAK-PBD to probe active Rac. Representative images show distribution of PAK-PBD and myosin II staining in control and TAPP2 KD cells. A multipolar distribution pattern was more often seen in TAPP2 KD cells, where both PAK-PBD and myosin II stain peripheral protrusions. The bar graph compares frequencies of the multipolar pattern (with more than two protrusions) in control and TAPP2 KD cells based on four experiments. For each group more than 100 cells were analyzed. The frequency is significantly higher in TAPP2 KD cells than in control cells, as indicated by Student’s *t* test: **p<0.01.

Rho family GTPases are important mediators of cell polarization events underlying cell migration. Typically, Rac is activated in the front to generate protrusions, while Rho is active in the back to promote retraction of the cell rear. To determine whether TAPP2 impacts on the activity of Rho family GTPases we performed pull-down assays to measure active Rac and Rho in control and TAPP2 KD cells in response to SDF-1 stimulation. TAPP2 KD cells exhibited over-activation of Rac1 but showed no significant difference in activity of Rho GTPases (Fig.5A). In the same experiments, the pull-down assays failed to detect active Cdc42 signal comparable to that of Rac or Rho, presumably due to its low abundance in these cells (data not shown). In an attempt to visualize the distribution of Rac activity, we used GST-tagged p21-binding domain of p21-activated kinase (PAK PBD) to probe active Rac. Cells were co-stained with antibody against non-muscle myosin II heavy chain, a widely used marker to label the back of migrating cells. TAPP2 KD cells showed a markedly altered staining pattern, with multiple protrusions stained with both anti-myosin and PAK PBD (Fig. 5B). The frequency of cells exhibiting this multi-polar pattern was significantly higher in TAPP2 KD cells than in control cells (Fig.5B right panel).

### TAPP2 KD Impairs Migration of Malignant B Cells into Bone Marrow Stromal Cell Layers

**Figure 6:**
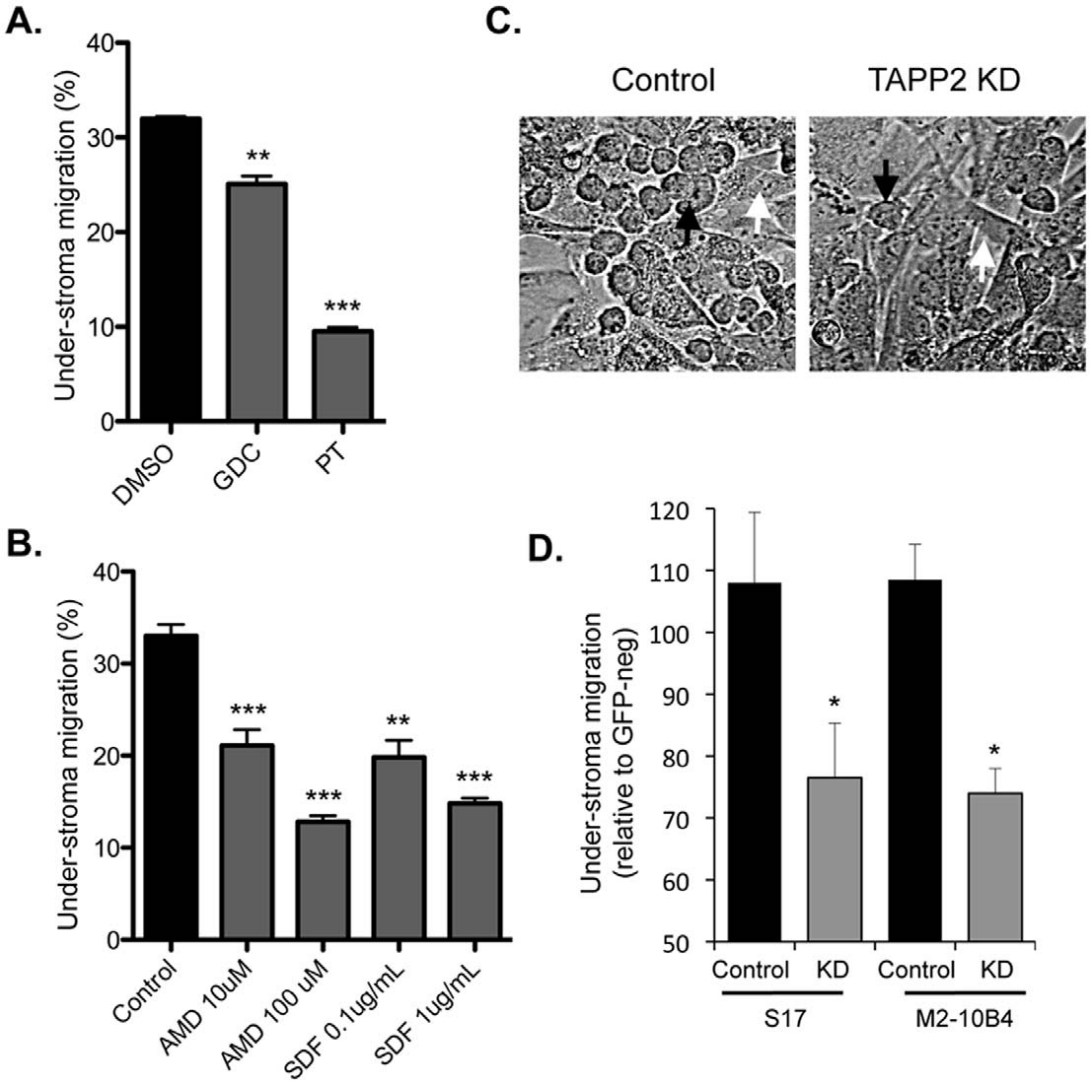
TAPP2 KD impairs leukemic cell migration into bone marrow stromal cell layers. NALM-6 cells were added to a confluent layer of S17 stromal cells and allowed to migrate for 10 hours. Under-stroma migration was quantified as percentage of total input cells as described in Methods. **A.** Under-stroma migration is pertussis toxin sensitive and partially blocked by PI3K inhibtion. PT: addition of 0.1 µg/ml pertussis toxin (GPCR inhibitor); GDC: addition of 2 µM GDC-0941 (pan PI3K inhibitor); DMSO, vehicle control for GDC-0941. **B.** Under-stroma migration is partially dependent on the SDF-1 receptor CXCR4. Control, not treated; AMD, 10 or 100 µM AMD3100 (CXCR4 inhibitor); SDF, 0.1 or 1 µg/ml SDF-1 (to desensitize CXCR4). Results are mean ± SD of three independent experiments. Significant difference in leukemic migration into the stromal layer was determined by Student’s *t* test: **p < 0.01 or ***p<0.001. **C.** Phase contrast imaging of the under-stroma migration of control or TAPP2 KD NALM-6 cells into S17 stromal cells. Migrated cells, indicated by black arrows, are characterized by the dark round appearance in contrast to the stromal cells, indicated by white arrows. Images represent two independent experiments. **D.** TAPP2 KD inhibits under-stroma migration. NALM-6 cells were partially transduced with GFP-expressing pSIH1-H1-copGFP control lentivirus or TAPP2 KD lentivirus. Migration into S17 or M2-10B4 stromal layers was normalized as the migration of GFP+ (transduced) cells relative to that of GFP- cells (internal control) in the same sample. Results from one experiment performed in replicates were shown as mean ± SD, representing two independent experiments confirming migration inhibition by TAPP2 KD. Significant difference between control and KD cells based on each stromal type was calculated with Student *t* test: *p<0.05.

Human malignant B cells are capable of migrating into layers of bone marrow stromal cells in a process also termed pseudoemperipolesis [34]. This chemokine-dependent process is thought to reflect a homing mechanism by which malignant B cells find favorable niches that promote their survival. To examine whether TAPP2 regulates this process we set up a co-culture system where NALM-6 cells migrated into layers of bone marrow stromal cells. We found that migration was inhibited by pertussis toxin, a GPCR inhibitor and to a lesser extent by PI3K inhibitor GDC-0941 (Fig. 6A), consistent with a chemokine receptor dependent event. Under-stroma migration was also partially inhibited by AMD3100, an antagonist of the SDF-1 receptor CXCR4, or by pretreatment with SDF-1 to downregulate CXCR4 surface expression (Fig. 6B). Consistent with the role of TAPP2 in SDF-1-dependent migration in transwell and microfluidic migration assays, TAPP2 KD also inhibited the under-stroma migration (Figs. 6B and 6C). These data suggested that disruption of TAPP2 function can impair malignant B cell migration in the context of interaction with chemokine-producing stromal cells.

## Discussion

Deciphering how cancer cells migrate is a major challenge but has important potential to guide development of new therapeutics. SDF-1 dependent migration is known to be critical for pathogenesis of many leukemias and lymphomas [1], [2] and drugs targeting the SDF-1 receptor are in development and clinical trials [3], [4]. Here we identified TAPP2 as a novel regulator of human cancer B cell migration in different contexts, including crawling on 2D substrates and passing through 3D micropores of the Transwell membrane. Migration into a bone marrow stromal cell layer, a process that depends on SDF-1 gradient, was also repressed in TAPP2 KD cells.

Our results provide initial insights into the mechanisms by which TAPP2 performs this function. TAPP2 was found to be directly implicated in chemokine-responsive changes in the actin cytoskeleton, a central machinery driving cell migration. Loss of TAPP2 dramatically altered the pattern of F-actin-based cellular protrusions induced by SDF-1, leading to loss of normal cellular polarity. Associated with this alteration was the over-activation and altered localization of active Rac, a master regulator of plasma membrane protrusion [21], [22]. In addition, a unique subcellular localization pattern of TAPP2 was revealed, which was not readily expected in a PI(3,4)P2-binding protein [35]. These data suggested that TAPP2 is important for regulation of F-actin dependent membrane protrusion and generation of cell polarity.

Cell migration relies on the precise front-back coordination of cellular events in order to establish functional migratory polarity. The current dominant model proposes that “frontness” signaling, controlled by Rac, promote membrane protrusion in the front but inhibit the formation of the back [36], [37]. Conversely, “backness” signaling pathways, such as those mediated by Rho promote the formation of the back but inhibit that of the front [36], [37]. Consistent with this mutual inhibition polarity model, the overactivation of Rac inhibits backness signaling in T lymphocytes, leading to multiple protrusions and loss of uropod (structure of migratory back in T cells) [38]. TAPP2 KD cells stimulated with SDF-1 showed mislocalized and increased activity of Rac and formation of multiple protrusions, consistent with failure to control “frontness” signaling and establish polarized “backness” necessary for effective migration.

We previously found that TAPP2, in association with the F-actin-binding proteins utrophin and syntrophin [39], [40], functions in the firm adhesion of BCR- or SDF-1-activated cancer B cells to extracellular matrix proteins [17]. Since control of adhesion and de-adhesion between a migrating cell and the substrate on which it moves is part of the known migration mechanisms, the TAPP2/utrophin complex may act at the level of adhesion to regulate Rac, membrane protrusion and migration. A recent study indicated that the rear formation of a migrating cell was characterized by the generation of stable adhesions that do not signal to Rac [41]. It is possible that TAPP2 KD disrupts adhesion-associated signaling pathways that restrict local Rac activity in regions that should form the rear of a polarized migratory cell, leading to frequent mislocalized membrane protrusions. More complete understanding of how TAPP2 executes such controls with relevance to the proper front-back polarity critical for migration will require future investigations.

TAPP2 is best known as a PI(3,4)P2 binding protein [14], [15], [16], and it would be expected that TAPP2 localization during cellular polarization would be controlled by the PI3K pathway. Several studies have found accumulation of PIP3 and PI(3,4)P2 at the front of migrating cells [21], [22], [35], [42]; however most of these studies used Akt-PH-EGFP probes, which bind both PIP3 and PI(3,4)P2. One report found accumulation of PIP3 but not PI(3,4)P2 in cAMP-stimulated migrating *Dictyostelium* cells [43]. Here we found that endogenous TAPP2 localized at the sides and rear of migrating cells rather than at the leading edge, suggesting either that PI(3,4)P2 does not accumulate at the leading edge in migrating B cells or PI(3,4)P2 is not the primary driver of TAPP2 localization in these cells. Given the co-localization of TAPP2 with its binding partner utrophin, it is possible that this interaction may primarily drive the localization and function of TAPP2 in migrating cells. Together with the finding that TAPP2 KD inhibited migration even when PI3K activity was inhibited, these observations suggested that the role of TAPP2 in chemokine-induced migration may be partially independent of PI3K-PI(3,4)P2 signaling.

A PI3K-independent role of TAPP2 in malignant B cell migration may represent new therapeutic opportunity. While PI3K inhibitor drugs have been demonstrated to strongly reduce the migration of malignant B cells in general [6], [9], complete inhibition remains to be accomplished [44], indicating the existence of alternative/redundant signaling mechanisms. In the context of PI3K-dependent migration, we found here that targeting both TAPP2 and PI3K achieved greater inhibition, which seems to reflect the shutting down of two independent components of migratory pathways. TAPP2 expression is largely restricted to hematopoietic cells with overexpression observed in more migratory CLL B cells [15], [16], [17]. Therefore, combining TAPP2 silencing and isoform-selective PI3K inhibitors could offer a relatively specific and more effective treatment targeting malignant B cell migration.

## Supporting Information

Figure S1 **TAPP2 regulates cell migration in other contexts. A.** Transwell migration of RS4;11 or RAJI cells to 100 ng/ml SDF-1 in the presence of drug vehicle control (DMSO) or PI3K inhibitors (2 µM) GDC-0941, CAL-101, AS-605240 or TGX-221. Bars are mean ± SD of three independent experiments. PI3K inhibition led to reduced migration in both cell lines as determined by Student’s *t* test: *p<0.05, **p < 0.01 or ***p<0.001. **B.** TAPP2 KD inhibits the migration of both RS4;11 and RAJI cells. TAPP2 KD and chemotaxis assay was performed in these cells using the same method as in Figure 1. Western blots are representative of two independent batches of transduction with TAPP2 shRNA expressing lentivirus. Results from one experiment are shown as mean ± SD of replicates, representing three independent experiments confirming migration inhibition by TAPP2 KD. Significance was determined with Student’s *t* test: *p<0.05. **C.** PI3K inhibitors and TAPP2 KD reduce basal motility of NALM-6 cells. Control (black) or TAPP2 KD (grey) NALM-6 cells were assayed for Transwell migration without chemokine induction in the presence of vehicle control (DMSO) or PI3K inhibitors (2 µM) GDC-0941, CAL-101 or TGX-221. Results are mean ± SD of three independent experiments. Significance of difference in migration was quantified by Student *t* test: *p<0.05.
(TIF)

Video S1 **Control B cell migration in a microfluidic system.** Control NALM-6 cells were loaded onto a microfluidic chemotaxis device and exposed to a 100 nM SDF-1 gradient (higher SDF-1 concentration at the bottom). Images were taken every 1 min for 4 hours and videos were generated from image stacks using ImageJ. Cell tracks are superimposed in the movies with blue tracks representing cells migration toward higher SDF concentration, and red tracks representing cells migrating away from higher SDF concentration.
(AVI)

Video S2 **TAPP2 KD B cell migration in a microfluidic system.** TAPP2 KD NALM-6 cell migration was recorded under the same conditions as Video S1.
(AVI)
