# Supplementary figures and images for: The Tandem PH Domain-Containing Protein 2 (TAPP2) Regulates Chemokine-Induced Cytoskeletal Reorganization and Malignant B Cell Migration
Source: PLoS One. 2013 Feb 27;8(2):e57809. doi: 10.1371/journal.pone.0057809 (PMC3583899; doi:10.1371/journal.pone.0057809)

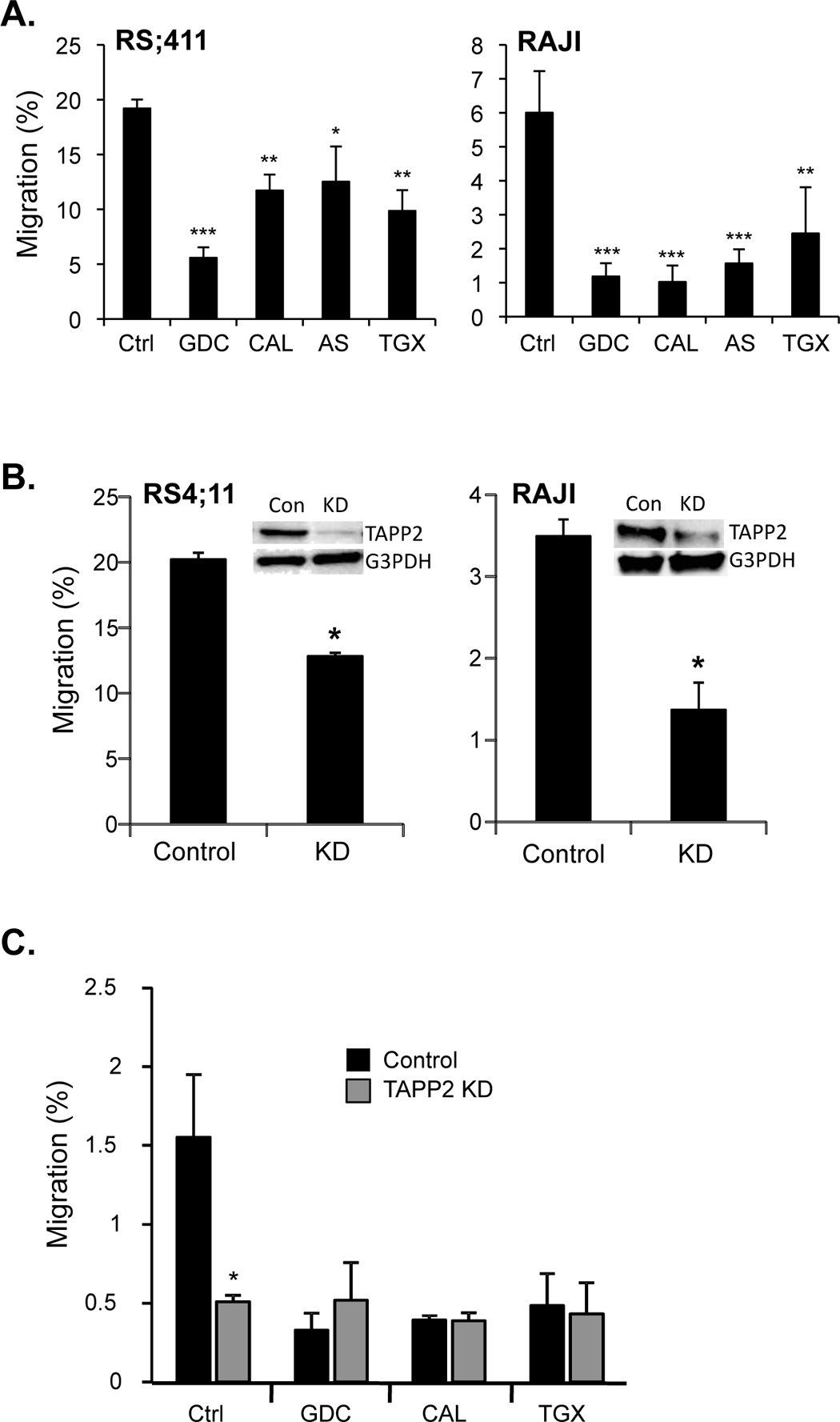

Supplement: Figure S1 — TAPP2 regulates cell migration in other contexts. A. Transwell migration of RS4;11 or RAJI cells to 100 ng/ml SDF-1 in the presence of drug vehicle control (DMSO) or PI3K inhibitors (2 µM) GDC-0941, CAL-101, AS-605240 or TGX-221. Bars are mean ± SD of three independent experiments. PI3K inhibition led to reduced migration in both cell lines as determined by Student’s t test: *p<0.05, **p < 0.01 or ***p<0.001. B. TAPP2 KD inhibits the migration of both RS4;11 and RAJI cells. TAPP2 KD and chemotaxis assay was performed in these cells using the same method as in Figure 1. Western blots are representative of two independent batches of transduction with TAPP2 shRNA expressing lentivirus. Results from one experiment are shown as mean ± SD of replicates, representing three independent experiments confirming migration inhibition by TAPP2 KD. Significance was determined with Student’s t test: *p<0.05. C. PI3K inhibitors and TAPP2 KD reduce basal motility of NALM-6 cells. Control (black) or TAPP2 KD (grey) NALM-6 cells were assayed for Transwell migration without chemokine induction in the presence of vehicle control (DMSO) or PI3K inhibitors (2 µM) GDC-0941, CAL-101 or TGX-221. Results are mean ± SD of three independent experiments. Significance of difference in migration was quantified by Student t test: *p<0.05. (TIF) [file pone.0057809.s001.tif]
